# A High Precision Approach to Calibrate a Structured Light Vision Sensor in a Robot-Based Three-Dimensional Measurement System

**DOI:** 10.3390/s16091388

**Published:** 2016-08-30

**Authors:** Defeng Wu, Tianfei Chen, Aiguo Li

**Affiliations:** 1 School of Marine Engineering, Jimei University, Xiamen 361021, China; 2 Fujian Provincial Key Laboratory of Naval Architecture and Ocean Engineering, Xiamen 361021, China; 3 School of Electrical Engineering, Henan University of Technology, Zhengzhou 450001, China; chen_tianfei@163.com; 4 School of Information Science and Technology, Dalian Maritime University, Dalian 116026, China; aiguoli_dmu@163.com

**Keywords:** robot based 3D measurement system, MLPNN, structured light vision sensor calibration, concentric circle

## Abstract

A robot-based three-dimensional (3D) measurement system is presented. In the presented system, a structured light vision sensor is mounted on the arm of an industrial robot. Measurement accuracy is one of the most important aspects of any 3D measurement system. To improve the measuring accuracy of the structured light vision sensor, a novel sensor calibration approach is proposed to improve the calibration accuracy. The approach is based on a number of fixed concentric circles manufactured in a calibration target. The concentric circle is employed to determine the real projected centres of the circles. Then, a calibration point generation procedure is used with the help of the calibrated robot. When enough calibration points are ready, the radial alignment constraint (RAC) method is adopted to calibrate the camera model. A multilayer perceptron neural network (MLPNN) is then employed to identify the calibration residuals after the application of the RAC method. Therefore, the hybrid pinhole model and the MLPNN are used to represent the real camera model. Using a standard ball to validate the effectiveness of the presented technique, the experimental results demonstrate that the proposed novel calibration approach can achieve a highly accurate model of the structured light vision sensor.

## 1. Introduction

Structured light vision sensors are widely applied in many fields, such as for three-dimensional (3D) measurements and quality control in manufacturing, because of their high measuring speed and appropriate accuracy. The sensor working modes can be roughly classified into two categories. One is a portable handheld mode based on self-positioning technology, such as Handyscan^®^ 3D scanners [1] and the ZScanner^®^ [2]. These handheld scanners are able to provide flexible and freeform off-line inspections. The other category is based on moving devices, such as coordinate measuring machines (CMMs) [3], industrial robots [4,5,6,7] or other specially designed mechanisms [8]. In regard to on-line inspection, the second category is suitable in this situation. The structured light sensor is fixed on the moving device, and the 3D information of the object part is obtained when it passes through the measuring range of the sensor. The obtained information is then compared with the original CAD model so that a closed-loop manufacturing process is formed; therefore, quality control is provided.

In the robot-based 3D measurement system, a structured light vision sensor is mounted on the arm of the robot. Calibrating the structured light vision sensor is a key aspect in this measurement system, as the system accuracy depends on the sensor calibration accuracy. This mainly consists of two steps. The first step is camera calibration; the second step is projector calibration. In the camera calibration step, two types of parameters, namely, the intrinsic parameters and the extrinsic parameters, should be calibrated. The extrinsic parameters denote the transformation relationships between the world coordinate frame, the camera coordinate frame and the image coordinate frame. The intrinsic parameters include the effective focal length, the lens distortion, the optical centre in the image plane and the length-width ratio of each pixel.

Existing camera calibration methodologies can be roughly classified into two categories. One is self-calibration (also known as 0D object-based calibration) methods and the other are object-based calibrations. Self-calibration [9,10,11,12,13] was first proposed by Maybank and Faugeras in [13]. Using this technique, the camera can be calibrated through point/line correspondences between images without a calibration object. This technique can be easily applied in uncontrolled environments where the geometry is unknown. However, one limitation of this approach is that a large number of parameters must be estimated so it is of low robustness and stability. Another limitation is that in regard to high accuracy 3D surface measurements, it is not adequate because of its relatively low accuracy compared with that of pre-calibration [14]. Object-based calibration utilizes a calibration object that has pre-known geometry information. The calibration object can be 3D, 2D or 1D. The 3D calibration object [15,16,17], which usually consists of two or three planes orthogonal to each other, is used to generate 3D calibration points. In this technique, the calibration object should be manufactured with high accuracy. Later, Zhang [18] proposed a flexible camera calibration technique that is based on a 2D calibration object. The calibration is achieved by viewing a plane from different unknown orientations, and high accuracy calibration points can be obtained. Recently, the use of a 1D calibration object for camera calibration has been proposed by many researchers [19,20,21,22]. As [20] has noted, in the 1D object based calibration technique, a very simple calibration object can be used to achieve a reasonable camera model without any pre-measurement.

In addition, it is well-known that circular features are widely adopted for camera calibration in computer vision. However, it should be noted that the centres of the projected circles are not exactly the projected centres of these circles. Therefore, many methods to obtain the real projected centres of the circles have been proposed. For instance, Heikkilä presented an iterative technique to obtain the real projected centres [23]. Later, Kim et al. [24] reported that the projected circle centre can be recovered accurately by using concentric circles. Xing et al. [25] proposed a novel approach to determine the real projected centres based on the theory of perspective projection and spatial analytical geometry using concentric circles. The method is simple and can be easily implemented in real experiments. In this study, a 2D target that consists of a set of concentric circles is designed, and sufficient calibration points are generated with the help of the robot. This procedure will be introduced in detail in the following section.

As far as the camera model is concerned, Tsai [26] proposed a two-stage calibration approach based on the radial alignment constraint (RAC). The radial distortion is considered in the method. Weng et al. [27] presented a camera model that accounts for all the major sources of camera distortion, namely, radial, decentring and thin prism distortions. Salvi et al. [28] compared many calibration techniques, including Tsai’s RAC two-stage approach [26] and the method of Weng et al. [27]. They concluded that the complete method in [27] does not achieve better accuracy than the simple iterative method modelling only radial distortion. In this study, only radial distortion is considered at first, and the division model (DM) [29,30] of the radial distortion is then adopted.

For projector calibration, the aim of this step is to find the relationship between the laser plane and the CCD array plane. Many methods [31,32,33,34,35] have been presented to determine the relationship between these two planes. In this study, the 3D world coordinate frame is chosen in the laser plane so that the laser plane equation can be easily obtained.

In addition to the aforementioned structured light vision sensor calibration technique, the artificial neural network technique [36,37] is also applied to solve the problem because of its strong non-linear approximation ability. For instance, Zhang and Wei [36] proposed an improved training algorithm for a multilayer perceptron neural network (MLPNN), and the improved MLPNN was successfully applied to calibrate a structured light vision sensor. The technique chose the world coordinate as the output of the network and the corresponding image coordinate as the input of the network so that the structured light vision sensor could be calibrated using the sole MLPNN.

In this study, a novel structured light vision sensor calibration technique is proposed. This technique combines the advantages of Tsai’s RAC two-stage method and the artificial neural network approach. To be specific, Tsai’s RAC two-stage method is first employed to generate an accurate calibration solution, and an MLPNN is then applied to identify the calibration residuals to achieve a much more accurate calibration result by compensating the residuals.

This paper is organized as follows: in Section 2, the robot-based 3D measurement system is introduced briefly. In Section 3, the camera model is given in detail. In Section 4, the camera calibration, including calibration point generation and the novel high precision calibration method are presented. The novel training algorithm for the MLPNN is also derived in this section. In Section 5, real experimental data are used to validate the effectiveness of the presented calibration method. Finally, some conclusions are given to summarize the study.

## 2. System Setup

**Figure 1:**
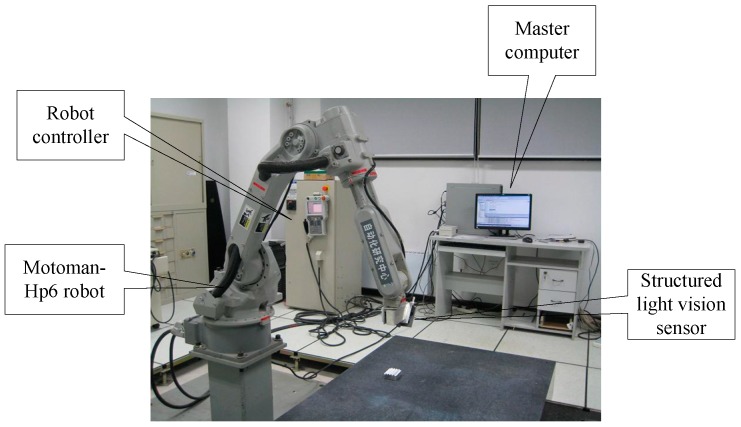
Robot-based 3D measurement system.

**Figure 2:**
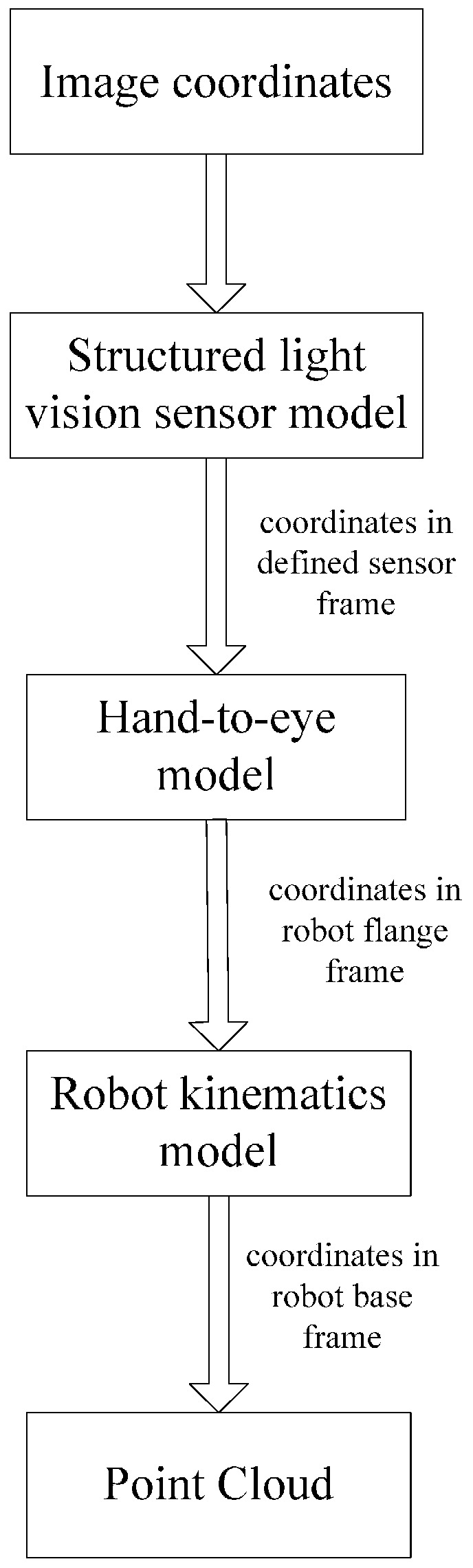
The presented 3D measurement system principle.

The robot based 3D measurement system is depicted in Figure 1, and it mainly consists of the following parts:

(1) Motoman-Hp6 robot;
(2) Structured light vision sensor [9]; Its specifications are as follows: measuring accuracy is smaller than 0.06 mm; measuring range is (90 mm, 190 mm); sampling speed is 12,000 pts/s; measuring depth of field is 100 mm;
(3) Master computer and measurement software system;
(4) Robot controller;

When a CMM-based 3D laser measurement system is used to scan a part, the measured object is difficult to scan without blind points when the scanned object surface is complex. The presented robot-based 3D measurement system is different from the CMM-based approach. In the new measuring approach, a measured object is placed in an experimental area at a standstill; all of the surface information of the object is obtained from once-off scanning because of the agility of the robot, which has six degrees of freedom while the CMM which just has three degrees of freedom. Because an adaptive structured light vision sensor developed by our group [9] is used, this system can measure an object to have profile containing the 3D coordinate information of the measured points. The 3D coordinate information of the measured points is obtained via the following stages: first, the laser emitted from the structured light vision sensor is projected on the object surface to form a light stripe. The distorted light stripe is captured by a CCD camera, and the 2D image coordinates of the obtained light stripe are calculated; Second, the 3D coordinate information in the defined vision sensor frame is obtained by the vision sensor model and the 2D image coordinates; Finally, the 3D coordinates in the robot base frame are determined from the hand-to-eye model [38] and robot kinematics model. The presented 3D measurement system principle is illustrated in Figure 2.

## 3. Camera Model

**Figure 3:**
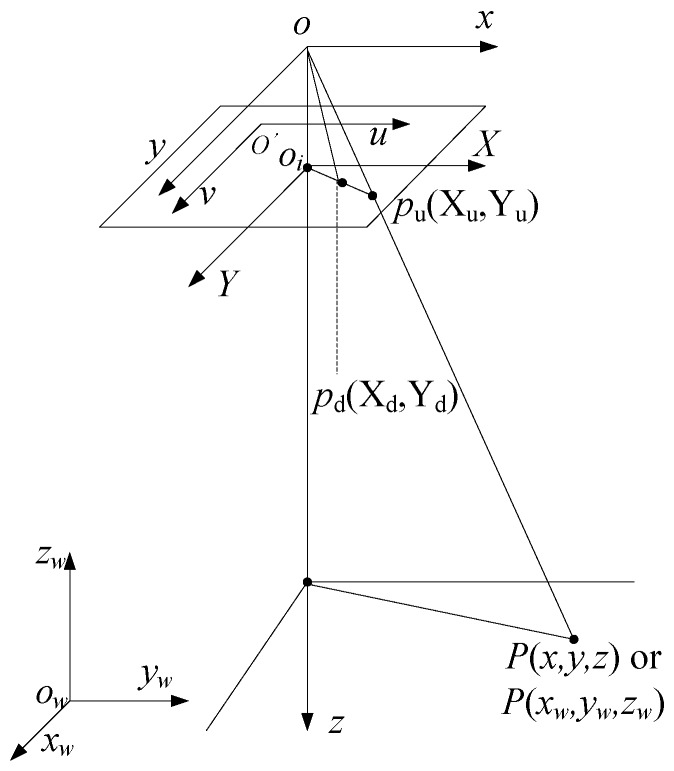
Principle of the perspective projection and camera model.

The camera model is described in [26]. The principle of the perspective projection and radial lens distortion is illustrated in Figure 3. There are three types of distortion: radial distortion, decentring and thin prism distortion. The radial distortion is first considered when establishing the camera model in this study because it is the main factor that affects the measurement accuracy.

In Figure 3, *o_w_,x_w_,y_w_,z_w_* is defined as the 3D world coordinate frame, *O_i_XY* is the CCD array plane coordinate frame, and *O_i_* is the intersection of the CCD array plane and the optical axis. *oxyz* is the 3D camera coordinate frame, where *o* is the projection centre of the camera, the *z* axis is the optical axis of the camera lens, and *x* and *y* are parallel to *X* and *Y*, respectively. *P* is a point in *oxyz* or *o_w_,x_w_,y_w_,z_w_*. Its corresponding point is *P_d_*(*X_d_,Y_d_*) instead of *P_u_*(*X_u_,Y_u_*) because of lens distortion. *f* is the effective focal length. *O’uv* is the computer image coordinate frame and *O’* is the origin of the image; the unit of the *u* axis and *v* axis is a pixel. Assume (*u*_0_,*v*_0_) be the coordinates of *O_i_* in *O’uv* and (*u*_0_,*v*_0_) is the principal point. The relationship between *o_w_,x_w_,y_w_,z_w_* and *O’uv* can be derived by the following steps:

The relationship between *oxyz* and *O_i_XY* is:

(1) $$\rho \left[\begin{array}{l} X\\ Y\\ 1 \end{array}\right]=\left[\begin{array}{ccc} f & 0 & 0\\ 0 & f & 0\\ 0 & 0 & 1 \end{array}\right]\left[\begin{array}{l} x\\ y\\ z \end{array}\right],$$

The transformation from *o_w_,x_w_,y_w_,z_w_* to *oxyz* is:

(2) $$\left[\begin{array}{c} x\\ y\\ z \end{array}\right]=R\left[\begin{array}{c} x_{w}\\ y_{w}\\ z_{w} \end{array}\right]+T,$$

where $R=\left[\begin{array}{ccc} r_{1} & r_{2} & r_{3}\\ r_{4} & r_{5} & r_{6}\\ r_{7} & r_{8} & r_{9} \end{array}\right]$, $Τ=\left[\begin{array}{c} T_{x}\\ T_{y}\\ T_{z} \end{array}\right]$, ***R*** is the rotation matrix and ***T*** is the translation vector.

The relationship between *O’uv* and *O_i_XY* is:

(3) $$\{\begin{array}{c} u=\frac{X}{dX}+u_{0}\\ v=\frac{Y}{dY}+v_{0} \end{array},$$

where *dX* and *dY* are the lengths of the pixel on the computer image.

Combining Equations (1)–(3), the camera model without lens distortion can be obtained as follows:

(4) $$\rho \left[\begin{array}{l} u\\ v\\ 1 \end{array}\right]=\left[\begin{array}{llll} \frac{fr_{4}}{dX}+r_{7}u_{0} & \frac{fr_{2}}{dX}+r_{8}u_{0} & \frac{fr_{3}}{dX}+r_{9}u_{0} & \frac{fT_{x}}{dX}+t_{x}u_{0}\\ \frac{fr_{4}}{dY}+r_{7}v_{0} & \frac{fr_{5}}{dY}+r_{8}v_{0} & \frac{fr_{6}}{dY}+r_{9}v_{0} & \frac{fT_{y}}{dY}+t_{y}v_{0}\\ r_{7} & r_{8} & r_{9} & T_{z} \end{array}\right]\left[\begin{array}{l} x_{w}\\ y_{w}\\ z_{w}\\ 1 \end{array}\right],$$

Taking the radial distortion into consideration, the polynomial model (PM) that is often used to describe radial distortion can be written as:

(5) $$\{\begin{array}{c} X=X_{d}(1+k_{1}r^{2}+k_{2}r^{4}+\cdots )\\ Y=Y_{d}(1+k_{1}r^{2}+k_{2}r^{4}+\cdots ) \end{array},$$

Fitzgibbon [29] suggested the DM as:

(6) $$\{\begin{array}{c} X=\frac{X_{d}}{\left(1+k_{1}r^{2}+k_{2}r^{4}+\cdots \right)}\\ Y=\frac{Y_{d}}{\left(1+k_{1}r^{2}+k_{2}r^{4}+\cdots \right)} \end{array},$$

Compared with the PM, DM is able to express high distortion at a much lower order. In this study, the single parameter DM is employed as suggested in [30]

(7) $$\{\begin{array}{c} X=\frac{X_{d}}{1+kr^{2}}\\ Y=\frac{Y_{d}}{1+kr^{2}} \end{array},$$

as the distortion model. In Equation (7), $r=\sqrt{X_{d}^{2}+Y_{d}^{2}}$ and *k* is the radial distortion coefficient.

In the above camera model with radial distortion (Equations (4) and (7)), the parameters *r*_1_~*r*_9_, *T_x_*, *T_y_*, *T_z_*, f, *u*_0_, *v*_0_ and *k* are unknown and need to be determined via camera calibration.

It should be noted that the camera model introduced here is a simple one. It doesn’t take every factor into the model like the model given in [34]. However, the camera model given in [26] can still be employed in some specific applications. The merit of the model in [26] is that it is easy to use for engineers. For high accuracy requirement, there are two possible directions: one is to take as many factors as possible into consideration just as the work proposed in [34]; the other one is that taking all of the factors that aren’t considered in the camera model into an ANN model and that is the approach taken in this paper.

## 4. Camera Calibration

### 4.1. Extraction of the Calibration Points

As mentioned before, concentric circles are used to generate the calibration points. Obtaining the real projective centre is based on the conclusion given in [25]:

*Conclusion*: The perspective projection of a concentric circle will produce two ellipses. A straight line will be obtained by the centres of the two ellipses, and the true concentric circle centre perspective projection is exactly on the line.

**Figure 4:**
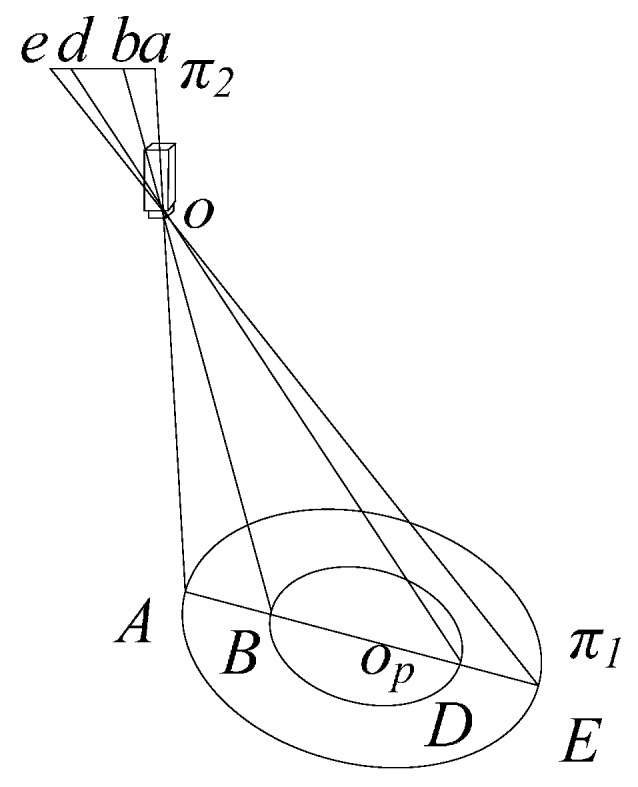
Concentric circle target.

Based on the above conclusion, a concentric circle calibration target is manufactured. The sub-pixel edges of the projected ellipses are then obtained via the Sobel+Zernike method [39]. The centres of the ellipses are calculated. If the distance between the two centres is smaller than a threshold value, i.e., 0.01 pixels, then the real circle projected centre is the average of the centres of the projected circles. Otherwise, the real circle projected centre will be determined via linear invariance and cross-ratio invariance. The procedure is as follows: first, establish the straight line equation from the two centres of the projected circles. Second, obtain the four points of intersection of the straight line and two projected circles and assume the points of intersection are *A*(*u_a_,v_a_*), *B*(*u_b_,v_b_*), *D*(*u_d_,v_d_*), and *E*(*u_e_,v_e_*). Furthermore, assume the real circle projected centre is *O*(*u_o_,v_o_*), as illustrated in Figure 4. The following equations can be obtained via linear invariance and cross-ratio invariance:

(8) $$\frac{u_{o}-u_{a}}{u_{o}-u_{b}}:\frac{u_{d}-u_{a}}{u_{d}-u_{b}}=\frac{R_{b}}{R_{s}}:\frac{R_{b}+R_{s}}{2R_{s}},$$

(9) $$\frac{v_{o}-v_{a}}{v_{o}-v_{b}}:\frac{v_{d}-v_{a}}{v_{d}-v_{b}}=\frac{R_{b}}{R_{s}}:\frac{R_{b}+R_{s}}{2R_{s}},$$

(10) $$\frac{u_{d}-u_{b}}{u_{d}-u_{o}}:\frac{u_{e}-u_{b}}{u_{e}-u_{o}}=\frac{2R_{s}}{R_{s}}:\frac{R_{b}+R_{s}}{R_{b}},$$

(11) $$\frac{v_{d}-v_{b}}{v_{d}-v_{o}}:\frac{v_{e}-v_{b}}{v_{e}-v_{o}}=\frac{2R_{s}}{R_{s}}:\frac{R_{b}+R_{s}}{R_{b}},$$

**Figure 5:**
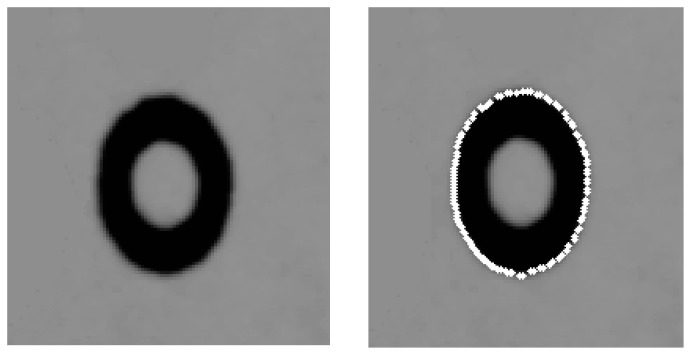
Concentric target (**left**) and its sub-pixel edge positioning result ((**right**), taken cylindrical for example).

**Figure 6:**
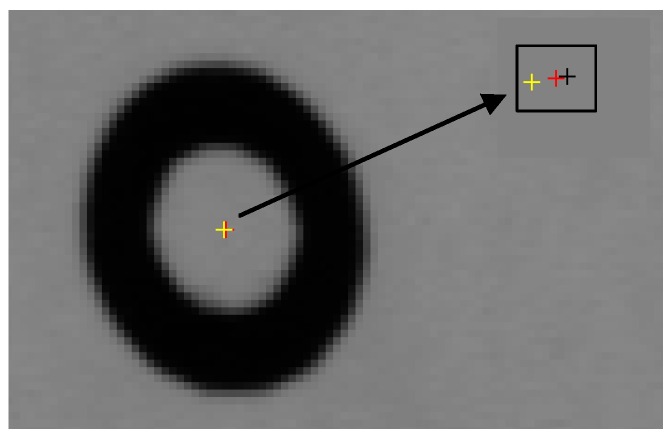
Circle centre projection result obtained by the concentric circle compensation method (the enlarged sub-image is framed at right top).

A sub-pixel edge detection based on an improved moment is presented in [39]. The presented approach in [39] is employed to determine the edge of the projected concentric circles. The experimental results are given in Figure 5. The real projected centre of the concentric circle is then obtained via the above procedure, and the result of one example is shown in Figure 6.

### 4.2. Solving Camera Model

The radial alignment constraint (RAC) two-stage method [26] is employed to solve the camera model. Equation (2) can be expressed as:

(12) $$\{\begin{array}{c} x=r_{1}x_{w}+r_{2}y_{w}+r_{3}z_{w}+T_{x}\\ y=r_{4}x_{w}+r_{5}y_{w}+r_{6}z_{w}+T_{y}\\ z=r_{7}x_{w}+r_{8}y_{w}+r_{9}z_{w}+T_{z} \end{array}$$

According to RAC, it follows that:

(13) $$\frac{X}{Y}=\frac{x}{y}=\frac{r_{1}x_{w}+r_{2}y_{w}+r_{3}z_{w}+T_{x}}{r_{4}x_{w}+r_{5}y_{w}+r_{6}z_{w}+T_{y}}$$

Changing the form of Equation (13) gives:

(14) $$\begin{array}{cc} \left[\begin{array}{ccccccc} x_{w}Y & y_{w}Y & z_{w}Y & Y & -x_{w}X & -y_{w}X & -z_{w}X \end{array}\right] & \\ \left[\begin{array}{c} r_{1}/T_{y}\\ r_{2}/T_{y}\\ r_{3}/T_{y}\\ T_{x}/T_{y}\\ r_{4}/T_{y}\\ r_{5}/T_{y}\\ r_{6}/T_{y} \end{array}\right] & =X \end{array}$$

Because *x_w_* = 0, it follows that:

(15) $$\left[\begin{array}{ccccc} y_{w}Y & z_{w}Y & Y & -y_{w}Y & -z_{w}X \end{array}\right]\cdot \left[\begin{array}{c} r_{2}/T_{y}\\ r_{3}/T_{y}\\ T_{x}/T_{y}\\ r_{5}/T_{y}\\ r_{6}/T_{y} \end{array}\right]=X,$$

According to Equation (15), an over-determined linear equations set is established, and the parameters in Equation (4) can be solved, except for *T_z_*, *f*, *k* and (*u*_0_,*v*_0_).

Two more equations can be found in Figure 3:

(16) $$\frac{X}{f}=\frac{x}{z}=\frac{r_{2}y_{w}+r_{3}z_{w}+T_{y}}{r_{8}y_{w}+r_{9}z_{w}+T_{z}},$$

(17) $$\frac{Y}{f}=\frac{y}{z}=\frac{r_{5}y_{w}+r_{6}z_{w}+T_{y}}{r_{8}y_{w}+r_{9}z_{w}+T_{z}},$$

Let *H_x_* = *r*_2_*y_w_* + *r*_3_*z_w_* + *T_x_*, *H_y_* = *r*_5_*y_w_* + *r*_6_*z_w_* + *T_y_* and *W* = *r*_8_*y_w_* + *r*_9_*z_w_*, so Equations (16) and (17) can be converted into:

(18) $$f\frac{H_{x}+T_{x}}{W+T_{z}}=X=\frac{X_{d}}{1+kr^{2}},$$

(19) $$f\frac{H_{y}+T_{y}}{W+T_{z}}=Y=\frac{Y_{d}}{1+kr^{2}},$$

Subtracting Equation (18) from Equation (19) and changing the form gives:

(20) $$\left[\begin{array}{ccc} H_{x}-H_{y}+T_{x}-T_{y} & r^{2}(H_{x}-H_{y}+T_{x}-T_{y}) & Y-X \end{array}\right]•\left[\begin{array}{c} f\\ fk\\ T_{z} \end{array}\right]=W(X-Y),$$

From Equation (20), *f*, *fk* and *T_z_* can be obtained by solving an over-determined linear equations set. Therefore, the parameters *T_z_*, *f* and *k* are obtained. (*u*_0_,*v*_0_) is the coordinate of the principal point. It is stated in [35] that the position of the principal point will cause a small calibration error when the distance between the principal point and the centre of the computer image is within the 20 pixel range. In this study, the centre of the computer image is first taken as the position of the principal point. A local search for (*u*_0_,*v*_0_) is then conducted by trial and error so that (*u*_0_,*v*_0_) is obtained.

### 4.3. Calibration Residuals Identification

**Figure 7:**
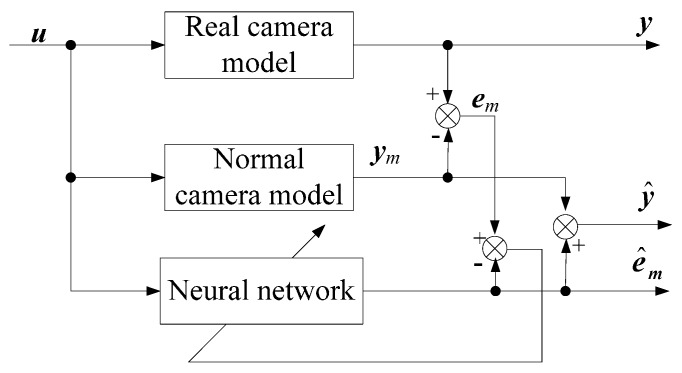
Structure of the proposed calibration method.

As we have mentioned before, although the camera model calibrated by Tsai’s RAC two-stage method is simple and can achieve high accuracy, some factors are not included in the model and this leads to the fact that there still exist calibration residuals that affect the measurement accuracy. These calibration residuals can be viewed as “unmodeled” part. In this paper, the calibration residuals are identified by an artificial neural network (ANN). The calibration residuals are then represented by the ANN model and compensated by the ANN so that the calibration accuracy is improved. The structure of the proposed calibration method is given in Figure 7. $\hat{y}$ and $\hat{z}$ are calculated by the camera model, where the relevant parameters are determined by the RAC two-stage method. It should be pointed out that Figure 7 should be highlighted and it is the main contribution of this study. General speaking, most of the calibration methods have either normal camera models or a neural network only. The proposed method shown in Figure 7 is a hybrid model of normal camera model and a neural network.

**Figure 8:**
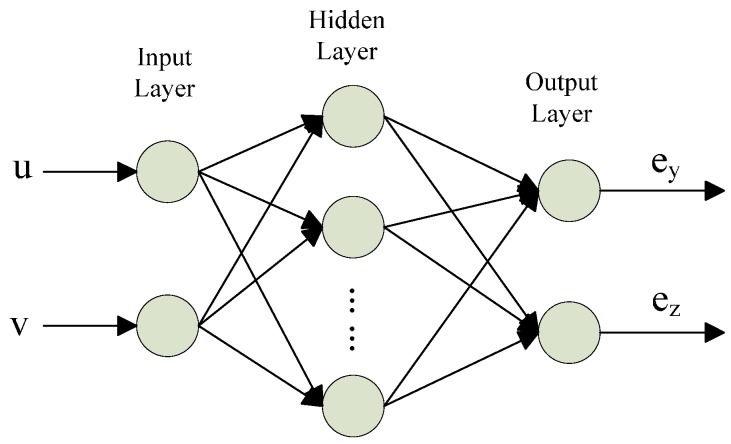
Architecture of the designed MLPNN.

MLPNN and RBFNN (radial basis function neural network) are two typical types of ANN for static modelling. Here, we choose the MLPNN to approximate the calibration residuals. A typical MLPNN mainly consists of three layers, namely, the input layer, the hidden layer and the output layer. The three layers are interconnected by weights. The designed architecture of an MLPNN is depicted in Figure 8. The input layer accepts elements of two-dimensional input data (*u*,*v*), which represent the coordinates of the image data. The second layer is composed of nonlinear functions to achieve a non-linear mapping. The output layer has two neurons that denote the e*y* and e*z*. e*y* is the residual between the obtained *y* coordinate and the RAC two-stage model output $\hat{y}$, and e*z* is the residual between obtained the *z* coordinate and the RAC two-stage model output $\hat{z}$.

The design of the MLPNN mainly consists of two aspects. One is determining a suitable number of hidden neurons, and the other is calculating the connection weights between the input/hidden and hidden/output layers. An MLPNN is incapable of differentiating between complex patterns, leading to only a linear estimate of the actual trend if there are too few neurons in the hidden layer. In contrast, if there are too many neurons in the hidden layer, the network will over fit the training data, leading to a poor generalization for the untrained data, and the training becomes time-consuming. The most popular method for finding the optimal number of hidden layer neurons is by trial and error, and this method is employed in this study. For the training algorithms of an MLPNN, there are also many approaches to train an MLPNN. The most widely used one is the so-called BP training algorithm. However, the BP cannot be guaranteed to find the global minimum of the error function because the gradient descent (GD) algorithm often falls into the local minimum area. Furthermore, the convergent rate becomes very slow in regard to later iterations. Therefore, many improved training algorithms are proposed to avoid the disadvantages of the gradient decent based back propagation algorithm. It should be noted that the training algorithm is not the main point discussed in this study. What we focus on is the application of the MLPNN in this novel calibration approach. In this study, the Levenberg-Marquardt (LM), scaled conjugate gradient (SCG), resilient (RP), one step secant (OSS), Conjugate gradient back-propagation with Fletcher-Reeves updates (CGF) and GD algorithms are adopted to train the designed MLPNN as these training algorithms are classical in MLPNN training, and comparisons are also made. It should be also noted that this paper focuses on the application of MLPNN instead of improvement of the training algorithm in MLPNN. Finally, the best training algorithm is selected.

## 5. Calibration Point Generation Procedure

**Figure 9:**
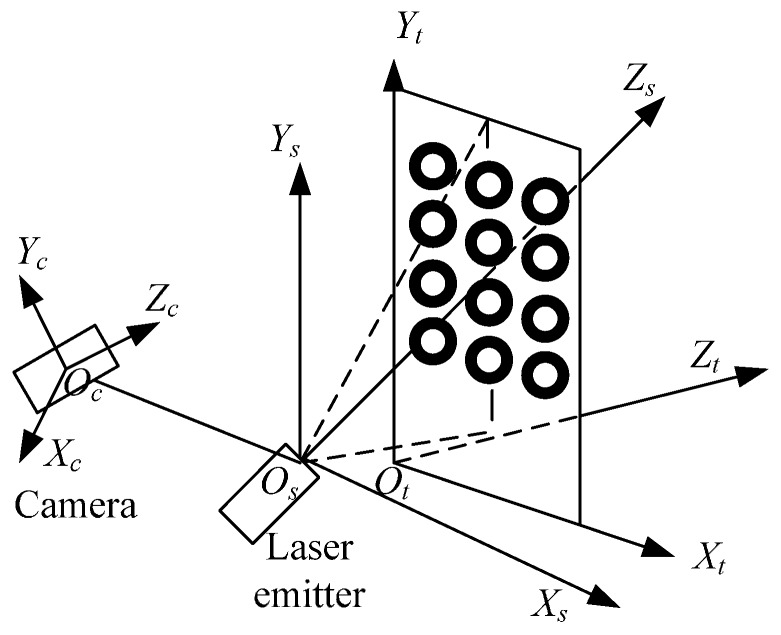
Sketch map for the defined coordinates of the sensor.

The six degree-of-freedom Motoman-HP6 robot is utilized to help generate calibration points because of its agility. It should be noted that the robot has been calibrated so that its positioning accuracy is improved and can be used in this situation. The structured light vision sensor mounted on the robot can be adjusted to any position in the robot workspace with any posture. With the above advantages, the relationship between the target coordinate frame *O_t_X_t_Y_t_Z_t_*, 3D camera coordinate frame *O_c_X_c_Y_c_Z_c_*, and sensor measurement coordinate frame *O_s_X_s_Y_s_Z_s_*, can be made to have only the translation part. The coordinate frame defined in calibration is shown in Figure 9.

**Figure 10:**
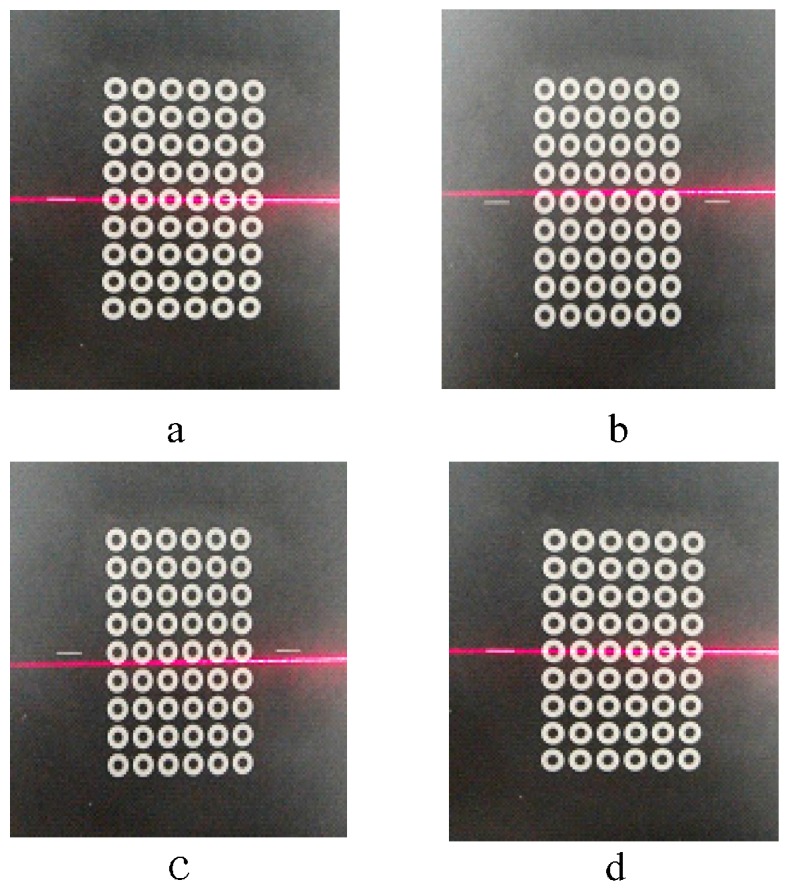
Alignment of the laser plane and the manufactured calibration line.

**Figure 11:**
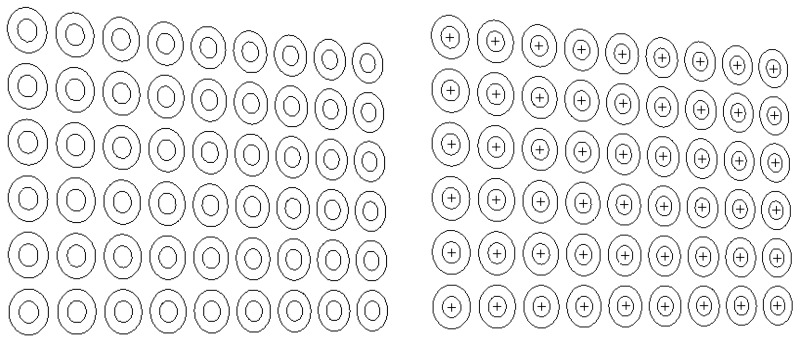
The corresponding calibration points (**Left**) Binarized image; (**Right**) Calibration points (Black cross line).

The calibration point extraction procedure is as follows:

*Step 1*: Make the robot end-effector move along its *z* axis when the robot is in its initial position. After the end-effector descends to a proper height, turn the laser on. Place the target on the fixed platform. The position of the target is chosen when the laser stripe covers the two auxiliary lines in the target, as depicted in Figure 10a.
*Step 2*: Make the robot end-effector move along its *z* axis continuously. If the laser stripe does not cover the two auxiliary lines, as shown in Figure 10b, the robot must be rotated along its *y* axis. The laser stripe will be emitted to the target, as illustrated in Figure 10c. Control the robot to move along its *x* axis to make the laser stripe cover the auxiliary lines again, as shown in Figure 10d.
*Step 3*: Repeat Step 2 until the laser stripe does not move away from the two auxiliary lines any more.
*Step 4*: Shut down the laser and make the robot move along its *z* axis. The robot translation distance along the *z* axis, obtained through the robot controller, can be taken as the *z* coordinate in the calibration measurement coordinates. Its *y* coordinate can be obtained by the exact distance between the calibration points. The corresponding points in the image coordinates (*u*,*v*) are obtained via the described procedure given in Section 4, as shown in Figure 11. From the above procedure, the calibration points will be generated in the measuring range.

## 6. Expermental Results

**Figure 12:**
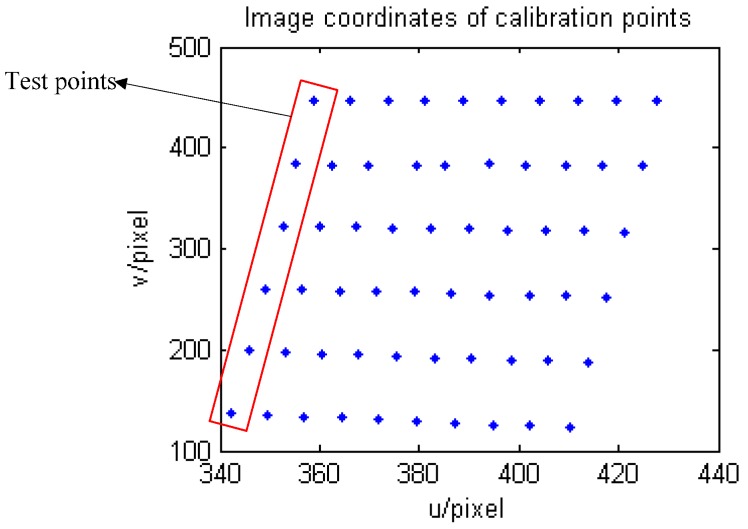
Calculated image coordinates (test points are framed in the red rectangle).

As we mentioned in the section above, the RAC two-stage method is first employed and an MLPNN is used to approximate the modelling residuals. The world coordinate (*yw*, *zw*) is selected as the output of the network, *k*, and its corresponding image coordinate (*u*,*v*) is used as the network input. The robot was made to move along its *z* axis 1 mm every step. Sixty calibration points were generated in terms of the proposed calibration point extraction procedure. Fifty-four points were selected to estimate all of the parameters in the camera model and were then used to train the designed MLPNN. The obtained parameters via the RAC method for the camera model are listed in Table 1. The six points left were used to test both the RAC two-stage method and the proposed novel approach. All of the obtained calibration points are depicted in Figure 12.

**Figure 13:**
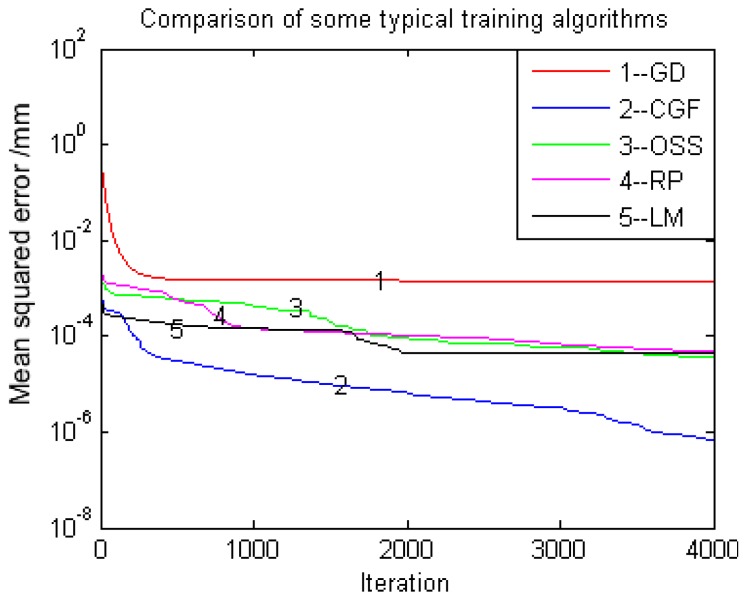
Performance of some typical training algorithms.

The training algorithms are an important part of MLPNN model development, and they were discussed in Section 4. Determining which training algorithm will be the fastest or most accurate for a given problem is a difficult task. An appropriate topology may still fail to give a better model unless it is trained by a suitable training algorithm. The trained neural network is used to represent the calibration residuals of the structured light vision sensor, which is mounted at the end of the Motoman robot in the proposed 3D measurement system. The different convergence performances of the five training algorithms are illustrated in Figure 13. It can be seen from the result that the CGF training algorithm has the best training performance over all of the other training algorithms because of its low training error and fast convergence rate in the training process, and it was selected to train the designed MLPNN.

**Figure 14:**
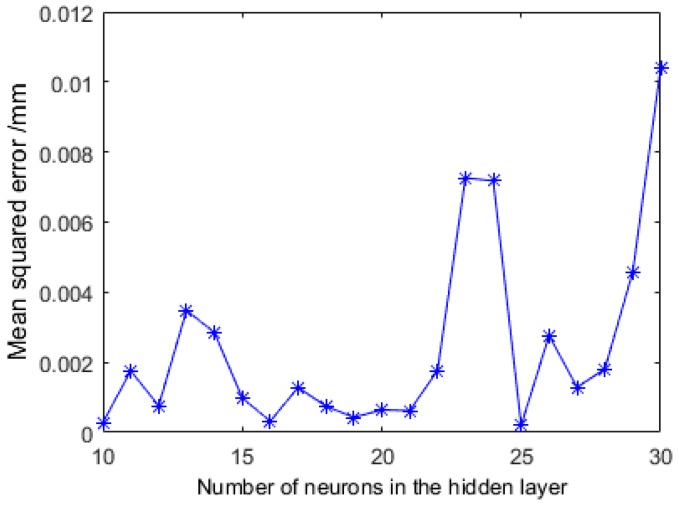
Relationship between the test error and number of neurons in the hidden layer.

The number of hidden layer neurons in the MLPNN was obtained by trial and error. In this study, the number of hidden layer neurons was changed from 10 to 30. The testing procedure was repeated 10 times (Note: this value was chosen empirically according to the number of neurons), and the average value is given in Figure 14. The results showed that the MLPNN calibration technique can perform well when the number of hidden layer neurons is 25.

The MLPNN with 25 neurons in the hidden layer was chosen and was trained for 4000 epochs, and as shown in Figure 14, after 4000 iterations, the training error remains the same for the CGF algorithm. The training process is within 1 minute offline. One of the best MLPNN structures was recorded. Then it was express as a mathematical function and implemented in the camera model. The calculation speeds of two methods are almost the same.

**Figure 15:**
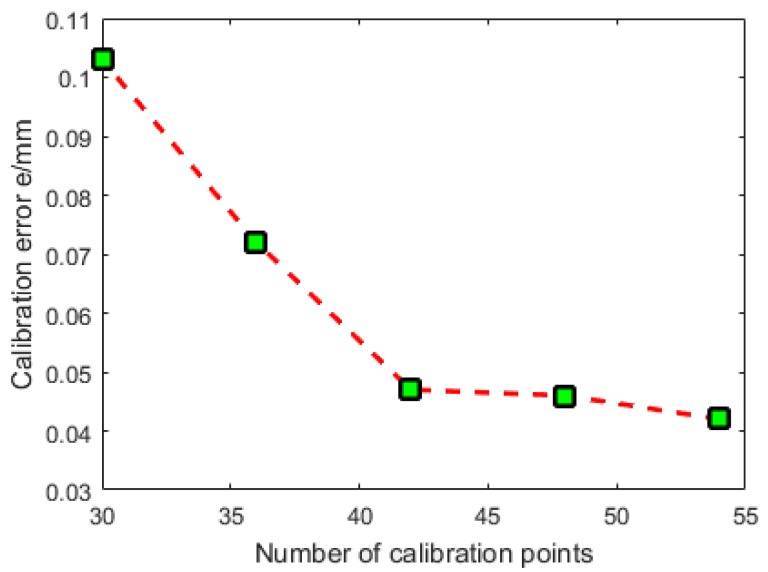
Relationship between number of calibration points and calibration error.

**Figure 16:**
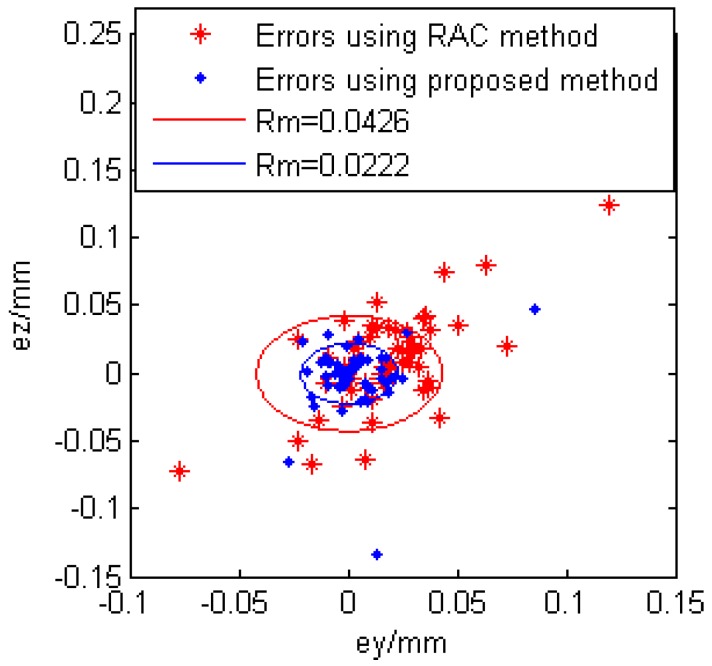
Error distribution from the image coordinates to the world coordinates.

There are many methods to evaluate the camera calibration accuracy. In [28], the camera accuracy evaluation methods are classified into two categories. The first one is based on analysing the discrepancy between the real position of the 3D object point with respect to the 3D position estimated from its 2D projection. The other one, however, is based on calculating the discrepancy between the real position, in pixels, of a 2D image point with the calculated projection of the 3D object point on the image plane. The first evaluation method is chosen in this study. Because the laser plane equation is *X_g_* = 0, it follows that the x coordinates of the calculated 3D object points are all 0. Generally speaking, the number of calibration points can be estimated by perspective projection equations. However, when the ANN is involved in the calibration model, it is difficult to estimate how many calibration points are good enough. In this study, the number of calibration points is changed and the calibration errors are recorded. The relationship between number of calibration points and calibration errors are shown in Figure 15. It follows that using 54 calibration points has best calibration performance. Then, the 54 calibration points are employed to evaluate the calibration accuracy and the calibration error is denoted as $e=\;\sqrt{e_{y}^{2}+e_{z}^{2}}$. The calibration error distribution is also illustrated in Figure 16. Using the traditional RAC calibration method, the maximum, mean and standard deviation of the radius of the calibration error are 0.2114 mm, 0.0426 mm and 0.0371 mm, respectively. After the novel approach is employed, the maximum, mean and standard deviation of the radius of the calibration error are 0.1541 mm, 0.0222 mm and 0.0291 mm, respectively. The calibration performance is improved after the application of the neural network.

The six remaining calibration points are then used to test the accuracy of the two calibration methods. The experimental results are listed in Table 2. From the obtained results, the average error is reduced from 0.0549 mm to 0.0403 mm. However, for each of the calibration points, all of the test accuracies are improved except the 2nd calibration point. It means that ANN doesn’t perform 100% good for the test data. However, the improvement rate is 5/6 = 83.3% in this case. If we look into the data in Table 2 further, it shows that two method perform closely with each other for the 2nd calibration point as the error is 0.0535 mm for RAC method while 0.0683 mm for proposed method. In other hand, the improvements for the rest 5 calibration points are much better. Table 2 also shows the performance of proposed method is much better than RAC method in a whole.

In addition to the camera calibration error, determining the laser plane equation as discussed before can also lead to a measurement error. After the RAC two-stage method parameters and MLPNN structure and parameters are determined, both of the calibrated structured light vision sensor models were implemented in the structured light vision sensor software system.

**Figure 17:**
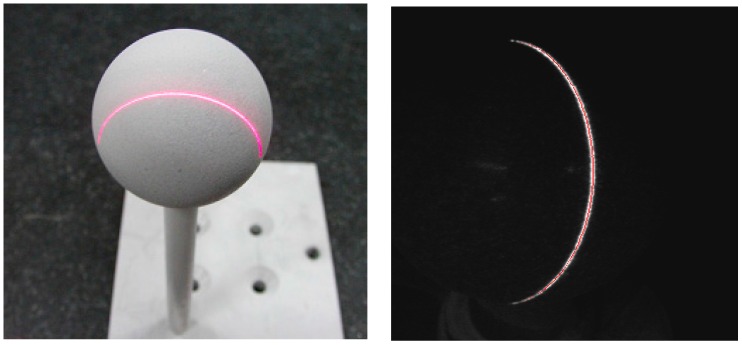
Standard ball measurement: standard ball (**left**) and the extracted projected laser stripe (**right**).

**Figure 18:**
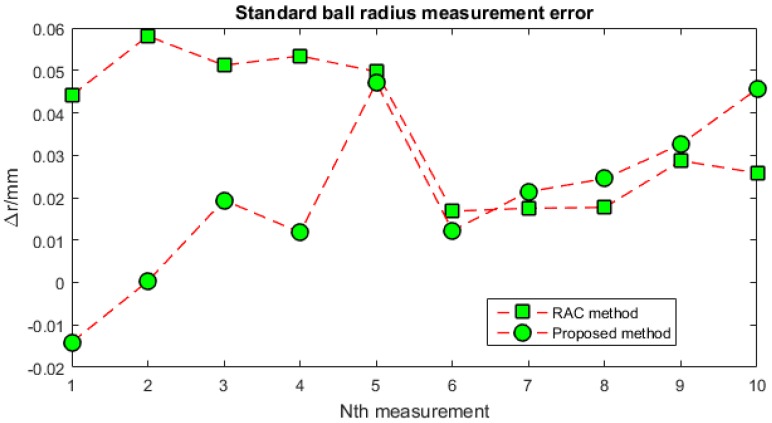
Standard ball radius measurement error of the sensor.

The measurement accuracy can be tested by measuring a standard ball with a known radius. The standard ball and its projected laser stripe are illustrated in Figure 17. The sensor is applied to measure the standard ball ten times, and each of the obtained radii is recorded for the RAC two-stage method and the presented method. All of the obtained values are listed in Table 3. The standard ball radius is (14.3005 ± 0.0028) mm, while *r* = 14.3005 mm is the nominal value and 0.0028 mm is the uncertainty. The measuring error is then denoted by Δr = *r_i_* − *r*. Both of the measuring errors for the two calibration methods are shown in Figure 18. From Table 3 and Figure 18, it can be seen that the average measuring accuracy is improved from 0.0368 mm to 0.0206 mm after the proposed calibration approach. It should be pointed out that there are all positive biases of RAC method, this is because that the camera model obtained via RAC method is not a completely model that takes as many factors as possible into consideration. However, there exists positive and negative bias in the proposed method as the model expressed by it is more accurate than RAC method as the powerful modelling of MLPNN. For the ten time measurements, the maximum measuring error is reduced from within 0.06 mm to within 0.05 mm.

## 7. Conclusions

A robot-based 3D measurement system has been established for 3D surface measurements, and it is hoped that the system will be applied for closed-loop manufacturing quality control in the near future. The measurement accuracy is one of the key components of this system. It is clear that the structured light vision sensor plays an important role in the presented system. To calibrate the vision sensor with much higher accuracy, a novel calibration point generation procedure, followed by a combination technique of the RAC method and the MLPNN approach, are presented. The novel calibration point generation procedure is of high accuracy, as the real circle projected centres are obtained via a set of concentric circles. The presented calibration approach is also of high accuracy because the MLPNN can compensate for the calibration residuals by the RAC method. The idea is inspired from adaptive control in control theory. This strategy can be employed in other measurement field applications. The experimental calibration results demonstrate the effectiveness of the presented method compared with the traditional RAC method. The calibration target employed in this study is simple and easy to manufacture. The experimental results show that a higher measuring accuracy can be obtained via the proposed novel calibration technique. The limitation of the proposed method is that a MLPNN should be designed and training data should be collected. In addition, the relationship between number of training data and precision of the calibration results and more comparisons should be further investigated in the future.

## Figures and Tables

**Table 1:** Calibration result of the normal camera model.

Coordinate of Principal Point (*u*_0_,*v*_0_)	Scale Factor *s_x_*	Focal Length *f* (mm)	Radial Distortion Coefficient *k* (pixel^−^^2^)	Rotation Matrix *R*	Transformation Matrix *T*
387, 305	0.96	11.6401	−0.0012	$\left[\begin{array}{ccc} -0.7782 & 0.0647 & -0.6247\\ -0.0655 & 0.9976 & 0.02173\\ 0.6246 & 0.0240 & 0.7806 \end{array}\right]$	$\left[\begin{array}{c} 23.81\\ -0.93\\ 107.81\; \end{array}\right]$

**Table 2:** Test camera calibration accuracy obtained by the RAC and proposed method.

No.	*u* (pixel)	*v* (pixel)	*y* (mm)	*z* (mm)	RAC Method			Proposed Method		
					*y*’ (mm)	*z*’ (mm)	*e* (mm)	*y*’ (mm)	*z*’ (mm)	*e* (mm)
1	342.2818	137.1483	10	9	9.9836	9.0386	0.0419	9.9754	9.0144	0.0285
2	345.6897	198.8738	5	9	4.9487	9.0155	0.0536	4.9317	8.9999	0.0683
3	349.1362	260.2563	0	9	−0.0299	8.9886	0.0320	0.0148	9.0079	0.0168
4	352.6949	322.7341	−5	9	−5.0806	8.9584	0.0907	−5.0466	8.9397	0.0762
5	355.3254	383.8221	−10	9	−10.0125	9.0492	0.0507	−9.9765	9.0169	0.0290
6	358.8092	446.0491	−15	9	−15.0493	9.0354	0.0606	−14.9817	9.0136	0.0229
**Ave.**							**0.0549**			**0.0403**

**Table 3:** Experimental result (for the standard ball measurement).

No.	Radius Measured by Vision Sensor after RAC Calibration/mm	Radius Measured by Vision Sensor after the Proposed Calibration Method/mm
1	14.3446	14.2864
2	14.3586	14.3008
3	14.3517	14.3199
4	14.3539	14.3123
5	14.3502	14.3476
6	14.3173	14.3127
7	14.3180	14.3219
8	14.3182	14.3250
9	14.3292	14.3331
10	14.3263	14.3461
**Average**	**14.3368**	**14.3206**
